# Characterization of Apicomplexan Amino Acid Transporters (ApiATs) in the Malaria Parasite Plasmodium falciparum

**DOI:** 10.1128/mSphere.00743-21

**Published:** 2021-11-10

**Authors:** Jan Stephan Wichers, Carolina van Gelder, Gwendolin Fuchs, Julia Mareike Ruge, Emma Pietsch, Josie L. Ferreira, Soraya Safavi, Heidrun von Thien, Paul-Christian Burda, Paolo Mesén-Ramirez, Tobias Spielmann, Jan Strauss, Tim-Wolf Gilberger, Anna Bachmann

**Affiliations:** a Centre for Structural Systems Biology, Hamburg, Germany; b Bernhard Nocht Institute for Tropical Medicine, Hamburg, Germany; c Biology Department, University of Hamburg, Hamburg, Germany; d Heinrich-Pette-Institute, Leibniz Institute for Experimental Virology, Hamburg, Germany; e GEOMAR Helmholtz Centre for Ocean Research Kiel, Kiel, Germany; f German Center for Infection Research (DZIF), partner site Hamburg-Borstel-Lübeck-Riems, Germany; University of Texas Southwestern

**Keywords:** *Plasmodium falciparum*, malaria, parasitology

## Abstract

During the symptomatic human blood phase, malaria parasites replicate within red blood cells. Parasite proliferation relies on the uptake of nutrients, such as amino acids, from the host cell and blood plasma, requiring transport across multiple membranes. Amino acids are delivered to the parasite through the parasite-surrounding vacuolar compartment by specialized nutrient-permeable channels of the erythrocyte membrane and the parasitophorous vacuole membrane (PVM). However, further transport of amino acids across the parasite plasma membrane (PPM) is currently not well characterized. In this study, we focused on a family of Apicomplexan amino acid transporters (ApiATs) that comprises five members in Plasmodium falciparum. First, we localized four of the P. falciparum ApiATs (*Pf*ApiATs) at the PPM using endogenous green fluorescent protein (GFP) tagging. Next, we applied reverse genetic approaches to probe into their essentiality during asexual replication and gametocytogenesis. Upon inducible knockdown and targeted gene disruption, a reduced asexual parasite proliferation was detected for *Pf*ApiAT2 and *Pf*ApiAT4. Functional inactivation of individual *Pf*ApiATs targeted in this study had no effect on gametocyte development. Our data suggest that individual *Pf*ApiATs are partially redundant during asexual *in vitro* proliferation and fully redundant during gametocytogenesis of P. falciparum parasites.

**IMPORTANCE** Malaria parasites live and multiply inside cells. To facilitate their extremely fast intracellular proliferation, they hijack and transform their host cells. This also requires the active uptake of nutrients, such as amino acids, from the host cell and the surrounding environment through various membranes that are the consequence of the parasite’s intracellular lifestyle. In this paper, we focus on a family of putative amino acid transporters termed ApiAT. We show expression and localization of four transporters in the parasite plasma membrane of Plasmodium falciparum-infected erythrocytes that represent one interface of the pathogen to its host cell. We probed into the impact of functional inactivation of individual transporters on parasite growth in asexual and sexual blood stages of P. falciparum and reveal that only two of them show a modest but significant reduction in parasite proliferation but no impact on gametocytogenesis, pointing toward dispensability within this transporter family.

## INTRODUCTION

Malaria parasites replicate within human erythrocytes during the asexual blood phase, which is responsible for the symptoms of the disease. Although Plasmodium falciparum is able to synthesize some amino acids *de novo* (1–6) during the intraerythrocytic development, the parasite relies heavily on amino acid acquisition from its host. Amino acids are derived either from the digestion of hemoglobin endocytosed from the infected erythrocyte (1, 7) or from the uptake of free amino acids from the blood plasma (1). Both processes are important for efficient parasite growth (8). The parasite is able to import all 20 naturally occurring α-amino acids from the external medium and uses them for its own protein synthesis (9–12). The import of isoleucine (13–15)—and for some P. falciparum strains methionine also (13)—is crucial for the survival of the parasite, as adult human hemoglobin lacks isoleucine. Accordingly, an increase in the permeability to a range of amino acids has been reported for erythrocytes upon *Plasmodium* infection (10, 11, 16, 17). This is mainly mediated via the new permeability pathways (NPPs) established by the parasite within the membrane of the infected erythrocyte (16, 17). While the subsequent transport across the parasitophorous vacuole membrane (PVM) is linked to nutrient-permeable channel activity (18–21), the molecular machinery responsible for the further transport across the parasite plasma membrane (PPM) is not well defined. Neutral amino acids like isoleucine and methionine traverse the PPM more rapidly than anionic and cationic amino acids, which may be coupled to the transport of other substrates like H^+^ or Na^+^ (16, 22). However, neither the membrane transporters nor the exact mechanism(s) by which amino acids cross the PPM have been characterized so far (8, 22).

The P. falciparum transportome is predicted to be encoded by 144 genes (23), of which at least 11 are classified as putative amino acid transporters (23, 24). Five of these putative amino acid transporters belong to the Apicomplexan amino acid transporter (ApiAT) (25, 26) family. This Apicomplexan-specific family of transmembrane transporters can be subdivided into the 11 subfamilies ApiAT1 to ApiAT11. Some of the subfamilies are lineage specific. For instance, ApiAT4, ApiAT8, ApiAT9, and ApiAT10 are present only in the genomes of *Plasmodium* spp. (1, 25). Others, such as the most ancient variant ApiAT2, can be found in many different Apicomplexan parasites (25). The main feature of the ApiAT family is the possession of multiple, typically 12, transmembrane domains characteristic of solute transporters with a signature sequence between transmembrane domains 2 and 3 (25). This classifies them as members of the major facilitator superfamily (MFS) (24, 27, 28). However, overall they have limited sequence similarity to other known eukaryotic or prokaryotic transporters (25).

P. falciparum possesses 5 of the 11 ApiAT subfamilies: P. falciparum ApiAT2 (*Pf*ApiAT2) (*Pf*ApiAT2/MFR4: PF3D7_0914700), *Pf*ApiAT4 (MFR5: PF3D7_1129900), *Pf*ApiAT8 (NPT1: PF3D7_0104800), *Pf*ApiAT9 (MFR2: PF3D7_0104700), and *Pf*ApiAT10 (MFR3: PF3D7_0312500), previously termed major facilitator superfamily-related transporter (MFR) or novel putative transporter (NPT). Previous work in the rodent malaria species Plasmodium berghei and the related apicomplexan parasite Toxoplasma gondii showed that P. berghei ApiAT8 (*Pb*ApiAT8) (or *Pb*NPT1) and several other T. gondii ApiATs (*Tg*ApiATs) are localized at the PPM (29–32) and possess amino acid transport activity (25, 26, 29–34). To date, only *Pb*ApiAT4 has been shown to play an important role in parasite proliferation within erythrocytes (35). Apart from this, *Pb*ApiAT8 is essential for gametocyte development (29, 30, 35), and all variants except *Pb*ApiAT2 appear to be necessary for exflagellation of male gametocytes (35). Additionally, sporozoite development likely requires *Pb*ApiAT2, *Pb*ApiAT4, *Pb*ApiAT9, and *Pb*ApiAT10 (35), and a lack of *Pb*ApiAT2 further impairs oocyst development. Furthermore, an association of episomally overexpressed *Pf*ApiAT10-GFP (*Pf*ApiAT10 tagged with green fluorescent protein [GFP]) (termed MFR3-GFP) with the mitochondrion has been reported (36).

In this study, we characterize the localization and essentiality of the ApiAT family members of P. falciparum during intraerythrocytic development in order to further dissect the function of these unique transporters.

## RESULTS

### P. falciparum ApiATs localize at the parasite plasma membrane.

**FIG 1:**
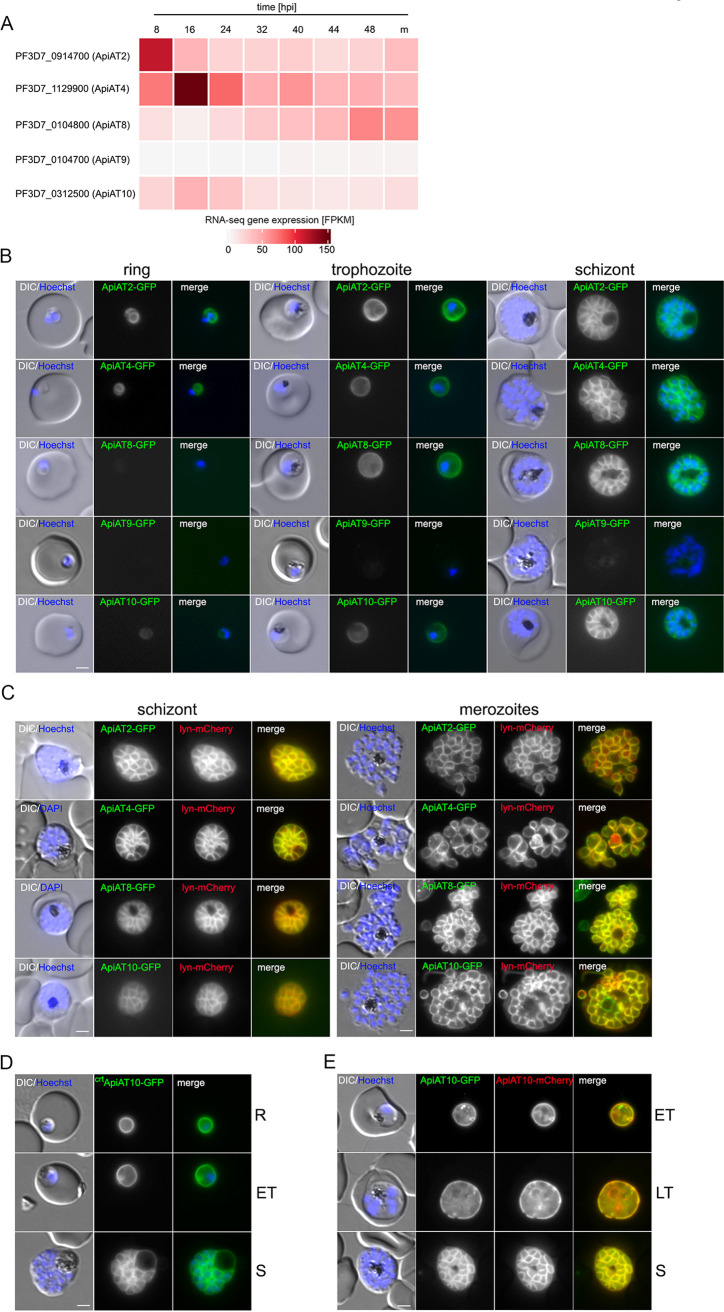
P. falciparum ApiATs localize to the parasite plasma membrane (PPM) during asexual blood stage development. (A) Heatmap of transcriptome sequencing (RNA-seq) gene expression profiles (37) of *Pf*ApiAT2, *Pf*ApiAT4, *Pf*ApiAT8, *Pf*ApiAT9, and *Pf*ApiAT10 during the asexual blood stage development. Time points are indicated as hours postinfection (hpi) plus merozoites (m). FPKM, fragments per kilobase per million. (B) Localization of *Pf*ApiAT2-GFP, *Pf*ApiAT4-GFP, *Pf*ApiAT8-GFP, *Pf*ApiAT9-GFP, and *Pf*ApiAT10-GFP by live-cell microscopy across the IDC of 3D7 parasites. DIC, differential interference contrast. (C) Colocalization of the GFP-tagged ApiAT fusion proteins with the PPM marker protein Lyn-mCherry in schizonts and merozoites. (D) Live-cell microscopy of 3D7-crt-ApiAT10-GFP parasites across the IDC. (E) 3D7-iGP-ApiAT10-GFP/ama1-ApiAT10-mCherry parasites at the trophozoite and schizont stage. Nuclei were stained with Hoechst-33342. Parasite stages are indicated as follows; ring stage (R), early trophozoite (ET), late trophozoite (LT) and schizont (S). Bars, 2 μm.

The five members of the ApiAT family in P. falciparum show different gene expression patterns and mRNA levels during the intraerythrocytic developmental cycle (IDC) (37). While *Pf*ApiAT2 has its maximum transcript level in early ring stage parasites (8 h postinfection [hpi]) and *Pf*ApiAT4 and *Pf*ApiAT10 mRNA levels peak in late ring stage parasites (16 hpi), *Pf*ApiAT8 shows a maximum of transcripts in late stage schizonts (48 hpi) and mRNA of *Pf*ApiAT9 is almost absent during the IDC (Fig. 1A). Overall, *Pf*ApiAT2 and *Pf*ApiAT4 are most abundantly expressed on the mRNA level.

To determine their protein expression and localization, we tagged each of the five members of the *Pf*ApiAT family endogenously with GFP using the selection-linked integration (SLI) system (38). Correct integration of the corresponding targeting plasmids into the respective genomic loci was verified by PCR (see Fig. S1A in the supplemental material). Except for 3D7-ApiAT9-GFP, all generated transgenic cell lines expressed the full-length fusion protein (Fig. S1B) to a sufficient level that allowed its subcellular localization. All of them are localized at the parasite periphery (Fig. 1B). Subsequent colocalization with the episomally expressed PPM marker Lyn (39)-mCherry (38) reveals PPM localization that becomes particularly evident in free merozoites, where PPM- and PVM-localized proteins can clearly be separated (Fig. 1C).

10.1128/mSphere.00743-21.1 FIG S1 PCR analysis and Western blots of 3D7-ApiAT-GFP and 3D7-ApiAT-TGD cell lines. (A) PCR-based analysis of unmodified wild-type (WT) and transgenic knock-in (KI) cell lines (3D7-ApiAT2-GFP-glmS, 3D7-ApiAT4-GFP-glmS, 3D7-ApiAT8-GFP, 3D7-ApiAT9-GFP, and 3D7-ApiAT10-GFP). Specific genomic modifications resulting from correct integration of the respective SLI-based vectors were tested targeting the 3′ and 5′ end of the locus. (B) Western blot analysis of wild-type (3D7) and knock-in (KI) cell lines (3D7-ApiAT2-GFP-glmS, 3D7-ApiAT4-GFP-glmS, 3D7-ApiAT8-GFP, and 3D7-ApiAT10-GFP) using mouse anti-GFP to detect the tagged full-length protein (upper panel) and rabbit antialdolase to control for equal loading (lower panel). The calculated molecular weight for each *Pf*ApiAT-GFP-fusion protein is indicated. Protein sizes are indicated in kilodaltons. (C) PCR-based analysis of unmodified wild-type (WT) and transgenic parasites modified by targeted gene disruption (TGD). Download FIG S1, TIF file, 0.9 MB. Copyright © 2021 Wichers et al. 2021 Wichers et al. https://creativecommons.org/licenses/by/4.0/ This content is distributed under the terms of the Creative Commons Attribution 4.0 International license.

In contrast to published data (36), we also found endogenously GFP-tagged *Pf*ApiAT10 localizing at the PPM. To reprobe into the apparent PPM localization of endogenously GFP-tagged *Pf*ApiAT10, we also overexpressed this gene as a GFP and mCherry fusion protein using two different promoters (*crt* [45] or *ama1* [46]). This allowed us to assess the influence of the tags as well as differential expression profiles on *Pf*ApiAT10 protein localization. All cell lines showed PPM localization of the *Pf*ApiAT10 fusion proteins (Fig. 1B to E and Fig. S1). Of note, functional inactivation of *Pf*ApiAT10 did not result in conferral of drug resistance to atovaquone (Fig. S2B).

10.1128/mSphere.00743-21.2 FIG S2 *Pf*ApiAT10 is PPM localized, and its functional inactivation has no influence on atovaquone sensitivity. (A) Live-cell microscopy of 3D7-iGP-ApiAT10-GFP parasites stained with MitoTracker Red CMXRos across the IDC and in gametocytes. Stages are indicated as follows: R, ring stage; ET, early trophozoite; LT, late trophozoite; ES, early schizont; LS, late schizont; G, gametocyte. Nuclei were stained with Hoechst-33342. Bar, 2 μm. (B) Drug susceptibility assays of 3D7-iGP and 3D7-iGP-ApiAT10-TGD parasites were performed with atovaquone (left) and dihydroartemisinin (DHA; right). Parasite growth was determined by measuring the DNA content using SYBR gold when exposed to various concentrations of drugs for 96 h. The growth of DMSO-treated control parasites was set at 100%. Shown are means ± SD of four or five independent biological replicates performed in technical duplicates. Calculated IC_50_ values with 95% confidence intervals are shown above each graph. Download FIG S2, TIF file, 0.9 MB. Copyright © 2021 Wichers et al. 2021 Wichers et al. https://creativecommons.org/licenses/by/4.0/ This content is distributed under the terms of the Creative Commons Attribution 4.0 International license.

### Individual *Pf*ApiATs are not essential for asexual blood stage development.

**FIG 2:**
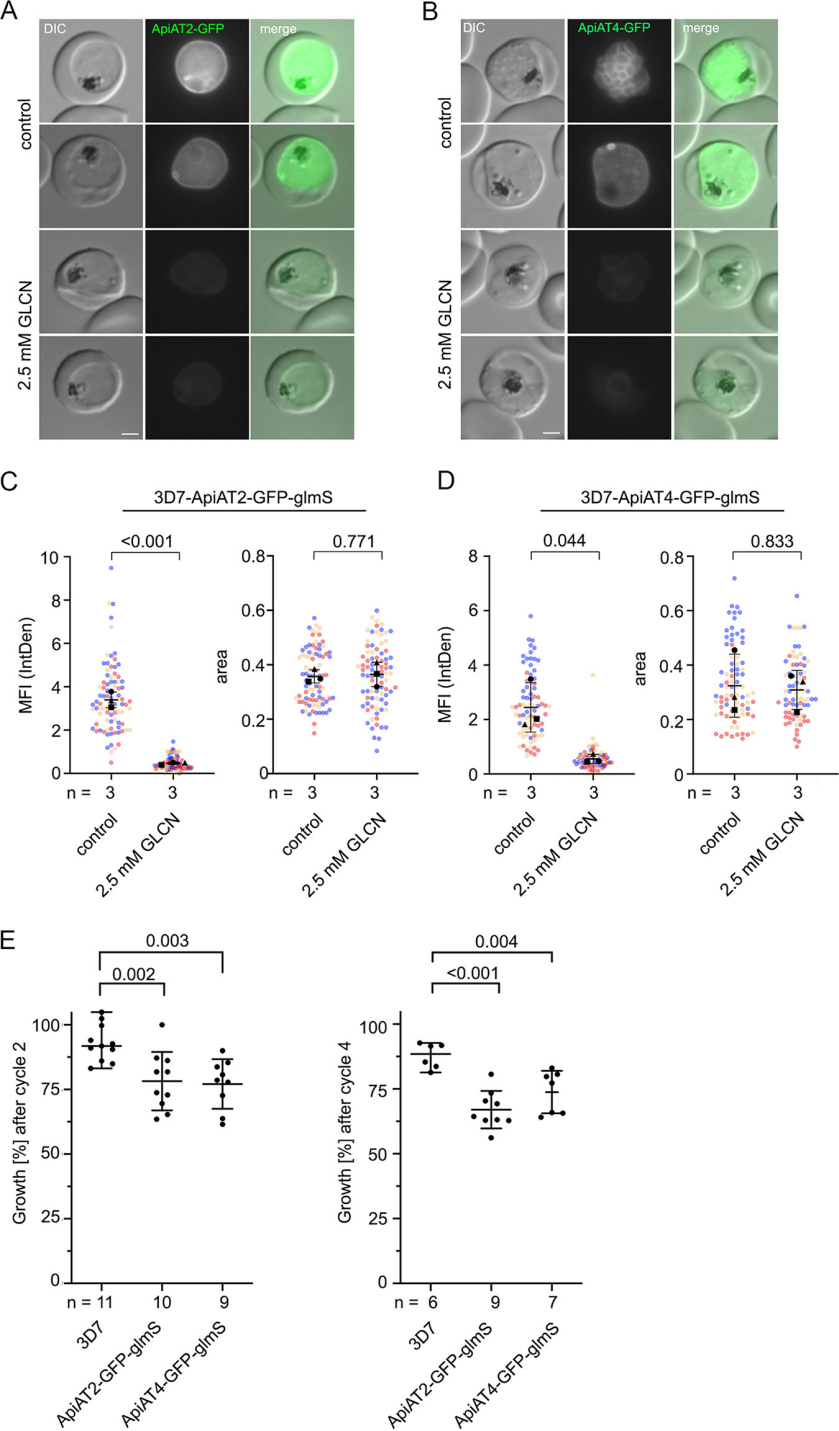
Conditional knockdown of *Pf*ApiAT2 and *Pf*ApiAT4 reveals a minor growth defect during asexual blood stage development. (A and B) Live-cell microscopy of 3D7-ApiAT2-GFP (A) and 3D7-ApiAT4-GFP (B) parasites, which were treated for 40 h with 2.5 mM glucosamine (GLCN) or that were left untreated (control). Bars, 2 μm. (C and D) Quantification of knockdown efficiency by measuring mean fluorescence intensity (MFI) density (IntDen, intensity density) and parasite size (area) of 3D7-ApiAT2-GFP (C) and 3D7-ApiAT4-GFP (D) parasites 40 h after treatment with or without 2.5 mM glucosamine. Data are displayed as means ± standard errors (SD) (error bars) of three independent experiments, and individual data points are displayed on a scatterplot color coded by experiments according to SuperPlots guidelines (82). *P* values displayed were determined using a two-tailed unpaired *t* test with Welch’s correction. (E) Growth of parasites treated with or without 2.5 mM GLCN after two and four parasite replication cycles as determined by flow cytometry. Shown are relative parasitemia values, which were obtained by dividing the parasitemia of glucosamine-treated cultures by the parasitemia of the corresponding untreated ones. Displayed are means ± SD of independent growth experiments with the number of experiments (*n*) indicated. Adjusted *P* values displayed were determined with a two-tailed unpaired *t* test with Welch’s correction and using the Benjamini-Hochberg correction afterwards accounting for multiple testing by comparing ApiAT2-GFP-glmS or ApiAT4-GFP-glmS cultured with 2.5 mM GLCN to 3D7 parasites cultured with 2.5 mM GLCN.

To probe into the essentiality of the ApiAT family for asexual parasite proliferation, we first targeted the most abundantly expressed *Pf*ApiATs, *Pf*ApiAT2 and *Pf*ApiAT4 by conditional knockdown. Downregulation was achieved by introducing a glmS ribozyme sequence (40) before the 3′ untranslated region (3′UTR) of either the *apiat2* or *apiat4* genomic locus (Fig. S1). The ribozyme was activated by the addition of 2.5 mM d-(+)-glucosamine hydrochloride (GLCN), which resulted in degradation of mRNA and therefore decreased protein levels. This was assessed and quantified by live-cell fluorescence microscopy after two cycles. GLCN treatment resulted in a decreased GFP fluorescence of 85.9% ± 0.9% (mean ± standard deviation [SD]) for 3D7-*Pf*ApiAT2-GFP or 74.8% ± 14.0% for 3D7-*Pf*ApiAT4-GFP (Fig. 2A to D) and led to a moderate, but significant, reduction of parasite growth of 14.0 to 20.7% compared to 3D7 parasites cultured with GLCN (Fig. 2E). These data indicate that *Pf*ApiAT2 and *Pf*ApiAT4 play a role in efficient blood cell proliferation but imply that they might be nonessential. Therefore, we targeted these genes with deletion constructs using the SLI system (38) that led to the expression of severely truncated versions of the ApiATs. In this targeted gene disruption (TGD) approach, we also included *Pf*ApiAT8 and *Pf*ApiAT10 (Fig. S1C).

**FIG 3:**
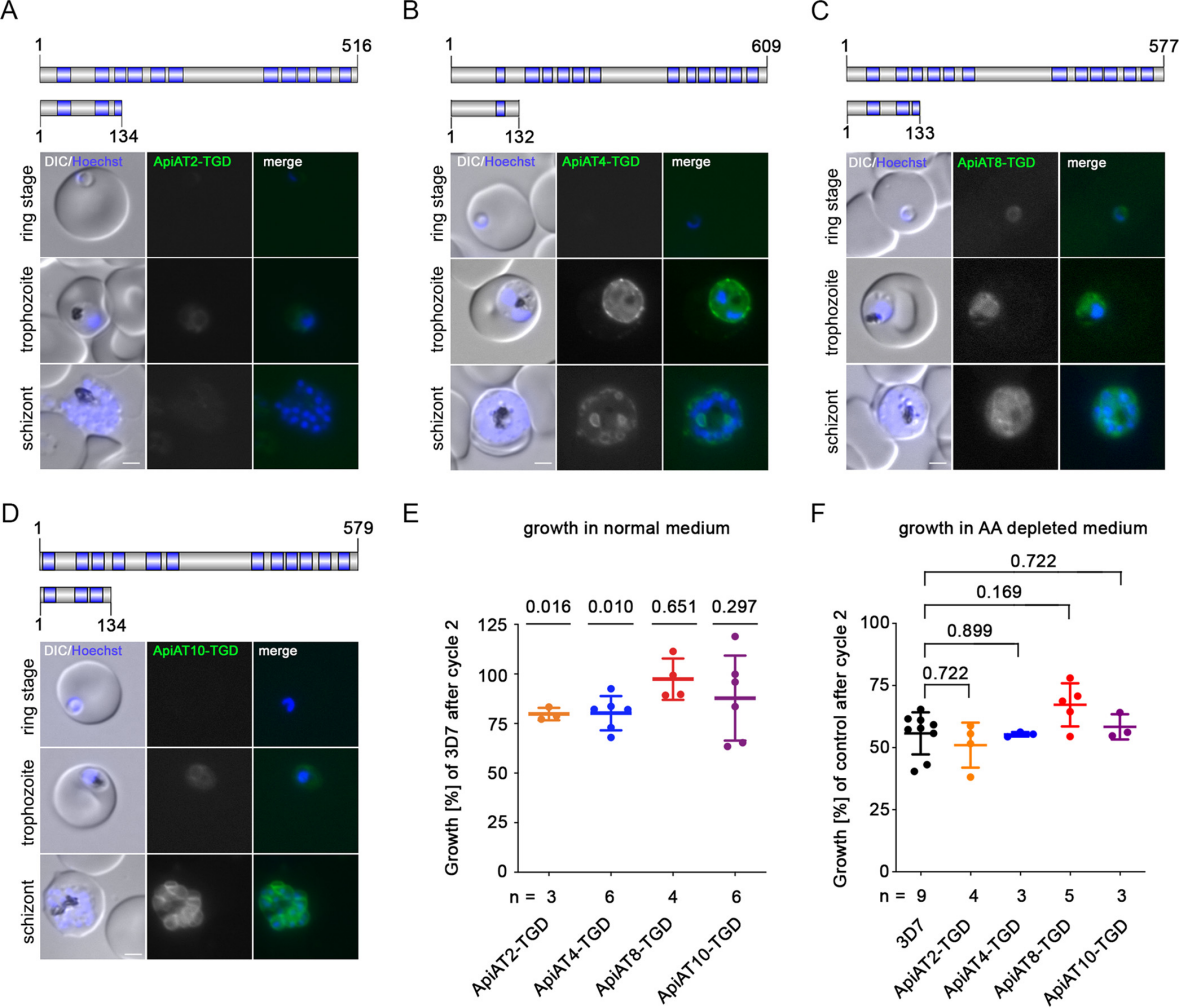
Targeted gene disruption (TGD) of *Pf*ApiAT2, *Pf*ApiAT4, *Pf*ApiAT8, and *Pf*ApiAT10 reveals the dispensability of *Pf*ApiATs for *in vitro* parasite proliferation. (A to D) Schematic representation of the full-length and truncated protein versions (top panel). Protein length (number of amino acids) and putative transmembrane protein domains (blue) are indicated. Localization of *Pf*ApiAT8-TGD-GFP (A), *Pf*ApiAT4-TGD-GFP (B), *Pf*ApiAT2-TGD-GFP (C), and *Pf*ApiAT10-TGD-GFP (D) in ring, trophozoite, and schizont stage parasites is shown in lower panels. Nuclei were stained with Hoechst-33342. Bars, 2 μm. (E) Growth of 3D7-ApiAT-TGD cell lines as a percentage of 3D7 parasite growth, monitored over two intracellular development cycles by flow cytometry. The number of independent growth experiments (*n*) per 3D7-ApiAT-TGD cell line is indicated. 3D7 wild-type parasites were measured in parallel. Statistical differences were analyzed using a one-sample *t* test with Benjamini-Hochberg correction accounting for multiple comparisons. (F) Growth of TGD and 3D7 cell lines cultivated in low-amino acid medium relative to their growth in standard medium is shown as percentage of growth after two parasite replication cycles. The number of individual growth experiments (*n*) performed is indicated for each 3D7-ApiAT-TGD line. Additionally, 3D7 wild-type parasites were analyzed with *n* = 9. No statistical differences were observed by comparing relative growth of TGD cell lines to 3D7 using a two-tailed unpaired *t* test with Bonferroni correction. AA, amino acid.

Imaging of the TGD cell lines revealed a more diffuse but still membrane-associated GFP signal (Fig. 3A to D). This might be due to the remaining transmembrane domains of the truncated *Pf*ApiAT mutants; however, our approach deleted at least three quarters of their predicted transmembrane (TM) domains and thus most likely abolished transporter activity. In concordance with the inducible knockdown data, functional inactivation by truncation of *Pf*ApiAT2 and *Pf*ApiAT4 in the corresponding transgenic cell lines (3D7-ApiAT2-TGD and 3D7-ApiAT4-TGD) led to a moderate decrease of parasite proliferation of 20.2% ± 3.2% and 19.8% ± 8.6% after two parasite replication cycles (Fig. 3E). No significant reduction of growth was observed upon disruption of *Pf*ApiAT8 and *Pf*ApiAT10 (Fig. 3E). Interestingly, cultivation in amino acid-depleted medium (approximately 90% reduced concentration) did not indicate a higher susceptibility of any of the TGD cell lines to low amino acid concentrations compared to wild-type parasites (Fig. 3F).

To probe into potential transcriptional perturbations within this gene family due to functional inactivation of a single member, quantitative real-time PCR (qPCR) analysis was performed using RNA from four different time points during asexual blood stage replication. However, no consistent upregulation of RNA levels of other *Pf*ApiAT family members was observed in individual *Pf*ApiAT TGDs (Fig. S3).

10.1128/mSphere.00743-21.3 FIG S3 Quantitative real-time PCR of individual *Pf*ApiAT-encoding genes and controls in 3D7-ApiAT-TGD and wild-type parasites. Expression of *apiat* genes and control genes (*sbp1*, *tom22*, *ama1*, and fructose-bisphosphate aldolase gene) normalized to the housekeeping control arginyl-tRNA synthetase gene over four different time points during the intraerythrocytic development cycle of P. falciparum parasites. Bars with individual measurements from *n* = 2 (3D7-ApiAT2-TGD and 3D7-ApiAT4-TGD) or *n* = 3 (3D7-ApiAT8, 3D7-ApiAT10, and 3D7 wild-type) biological replicates. Gene accession numbers, primer sequences, and amplification efficiencies are listed in Table S1 in the supplemental material. Download FIG S3, TIF file, 0.5 MB. Copyright © 2021 Wichers et al. 2021 Wichers et al. https://creativecommons.org/licenses/by/4.0/ This content is distributed under the terms of the Creative Commons Attribution 4.0 International license.

### *Pf*ApiATs are dispensable during gametocyte development.

**FIG 4:**
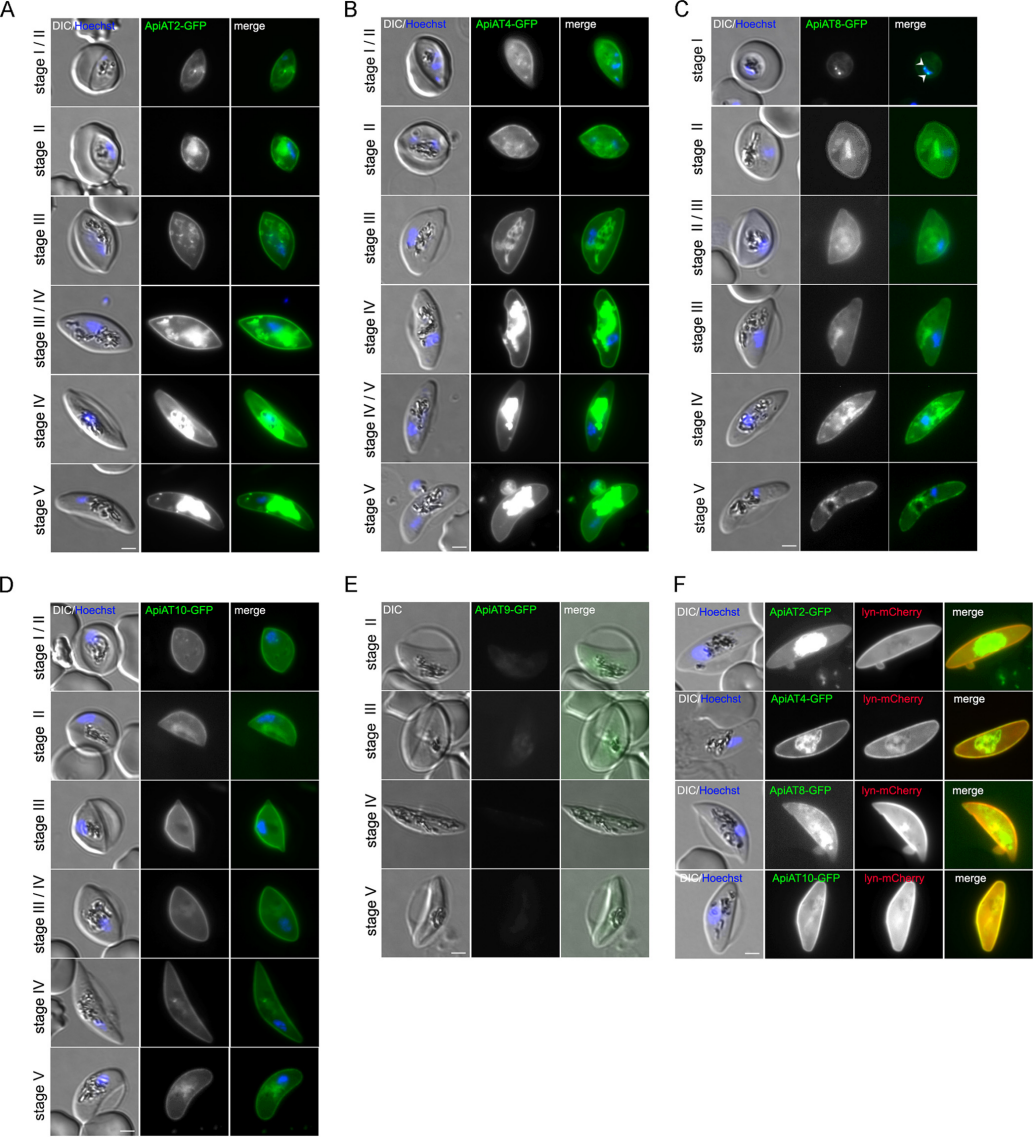
P. falciparum ApiATs localize to the parasite plasma membrane (PPM) during gametocyte development. (A to D) Localization of *Pf*ApiAT2-GFP (A), *Pf*ApiAT4-GFP (B), *Pf*ApiAT8-GFP (C), and *Pf*ApiAT10-GFP (D) in individual 3D7-iGP-ApiAT-GFP cell lines during gametocyte development (stages I to V) as determined by live-cell microscopy. White arrowheads indicate remaining GDV1-GFP signal observed in close proximity to the Hoechst signal in the 3D7-iGP-ApiAT8-GFP cell line (C) as previously described (60, 83, 84). (E) Localization of *Pf*ApiAT9-GFP during gametocytogenesis was assessed with the 3D7-ApiAT9-GFP cell line (see Fig. 1) upon induction with choline depletion. (F) Colocalization of the GFP-tagged ApiAT fusion proteins with the PPM marker protein Lyn-mCherry. Nuclei were stained with Hoechst-33342. Bars, 2 μm.

**FIG 5:**
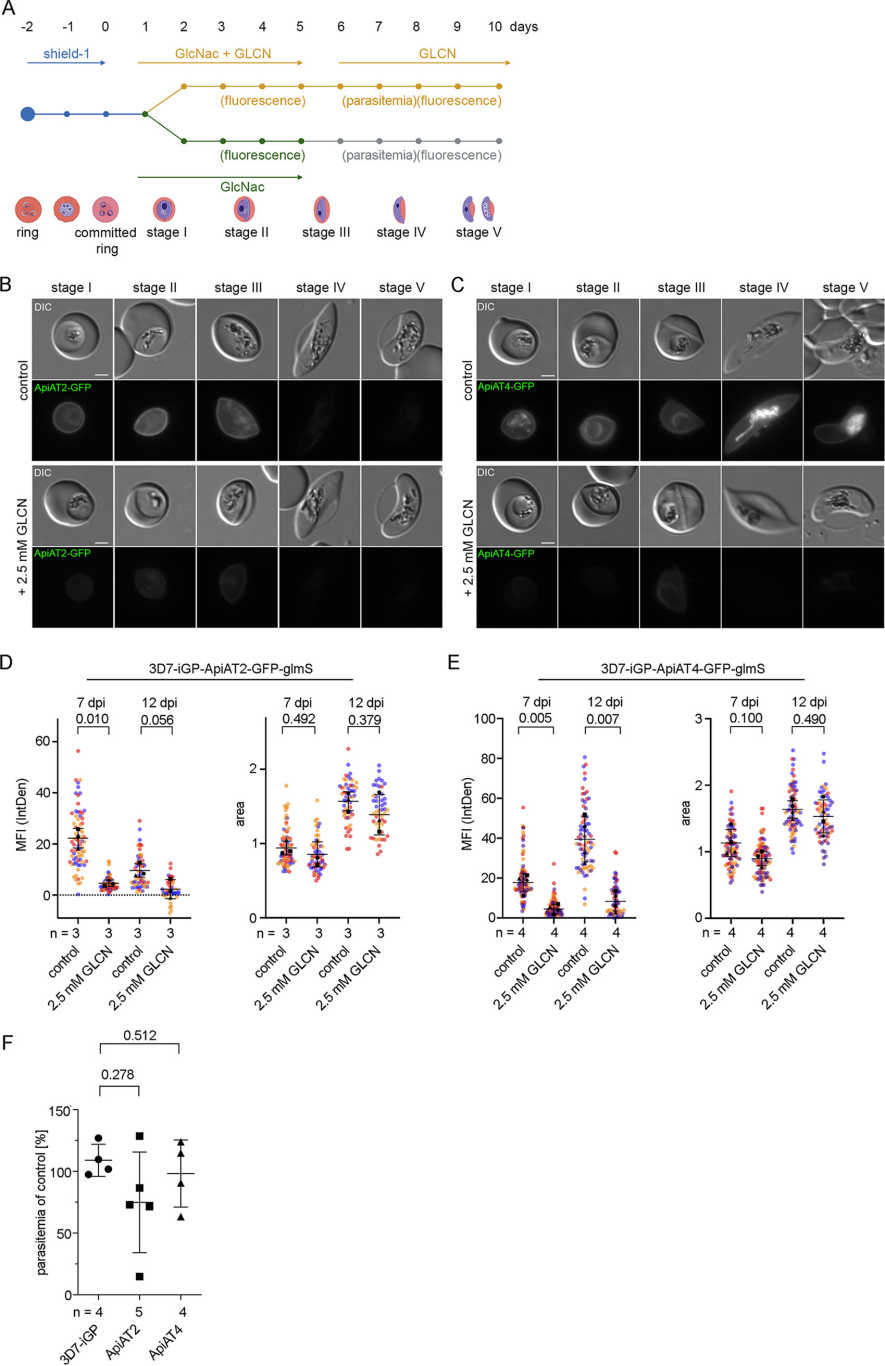
Conditional knockdown of *Pf*ApiAT2 and *Pf*ApiAT4 reveals dispensability for gametocyte development. (A) Schematic representation of the experimental setup. (B and C) Live-cell microscopy of parasites with identical settings of 3D7-iGP-ApiAT2-GFP (B) and 3D7-iGP-ApiAT4-GFP (C) stage I to V gametocytes. Bars, 2 μm. (D and E) Quantification of knockdown by measuring mean fluorescence intensity (MFI) density and size (area) of 3D7-iGP-ApiAT2-GFP (D) and 3D7-iGP-ApiAT4-GFP (E) parasites at day 7 and day 12 postinduction of gametocytogenesis cultured either with or without (control) 2.5 mM GLCN. Data are displayed as means ± SD of three (3D7-iGP-ApiAT2-GFP) or four (3D7-iGP-ApiAT4-GFP) independent experiments, and individual data points are displayed on a scatterplot color coded by experiments according to SuperPlots guidelines (82). *P* values displayed were determined with a two-tailed unpaired *t* test with Welch’s correction. (F) For each condition, gametocytemia at day 10 post-gametocyte induction was determined by counting between 702 and 7,693 (mean, 2,210) cells per condition in Giemsa-stained thin blood smears. The relative gametocytemia values (as a percentage) displayed were obtained by dividing the gametocytemia of glucosamine-treated cultures by the gametocytemia of the corresponding untreated cultures. Displayed are means ± SD of independent growth experiments with the number of experiments (*n*) indicated. A two-tailed unpaired *t* test with Welch’s and Benjamini-Hochberg correction was used to calculate multiplicity-adjusted *P* values for ApiAT2-GFP-glmS or ApiAT4-GFP-glmS versus 3D7-iGP parasites all cultured with 2.5 mM GLCN.

**FIG 6:**
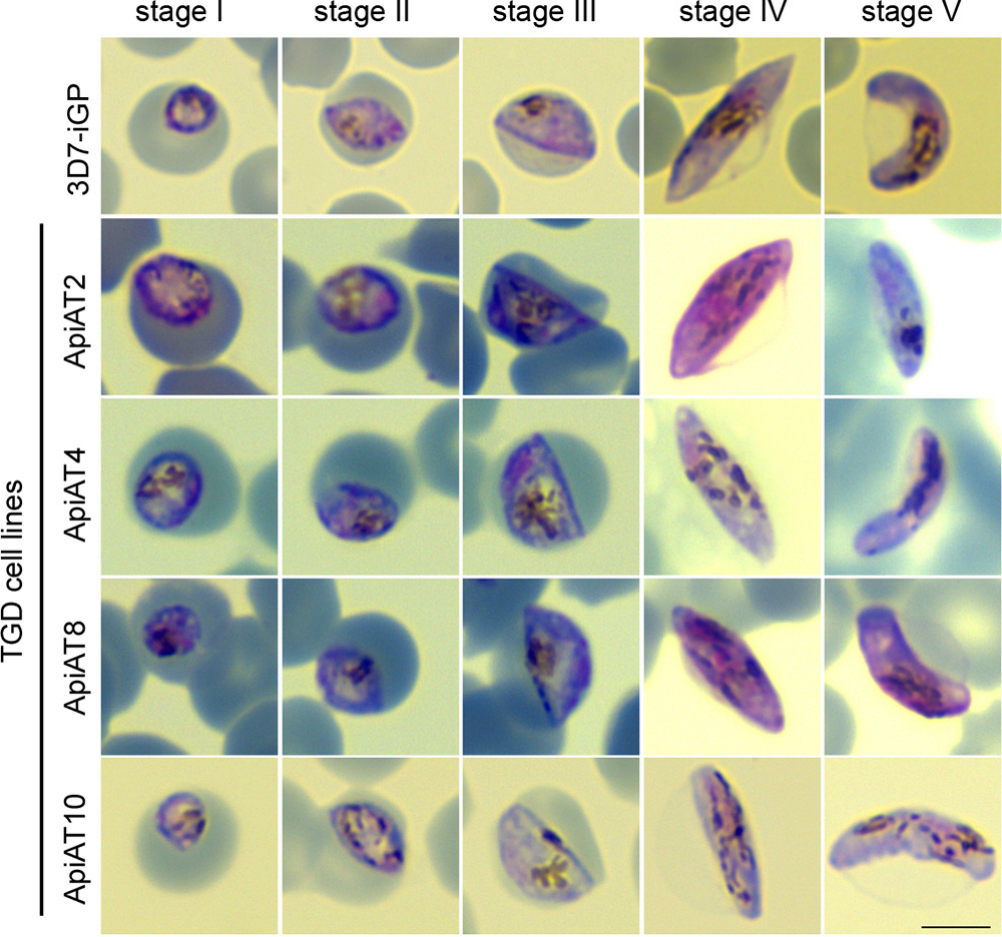
Targeted gene disruption (TGD) of *Pf*ApiAT2, *Pf*ApiAT4, *Pf*ApiAT8, and *Pf*ApiAT10 reveals dispensability of ApiATs for gametocyte development. Representative images from two (*Pf*ApiAT2 and *Pf*ApiAT4) or three (*Pf*ApiAT8 and *Pf*ApiAT10) independent experiments derived from Giemsa-stained thin blood smears of gametocyte stages I to V of 3D7-iGP, 3D7-iGP-ApiAT2-TGD, 3D7-iGP-ApiAT4-TGD, 3D7-iGP-ApiAT8-TGD, and 3D7-iGP-ApiAT10-TGD parasites. Bar, 5 μm.

Previous data (30, 35) indicated a role of ApiAT8 during gametocyte development of the rodent malaria parasite P. berghei. Therefore, we reengineered the GFP-tagged gene knockdown (Fig. S4) and deletion cell lines (Fig. S5) for *Pf*ApiAT2, *Pf*ApiAT4, *Pf*ApiAT8, and *Pf*ApiAT10 in an inducible gametocyte-producing parasite line (3D7-iGP-GDV1GFP-DD [41]) using the same SLI approach. The resulting parasite lines allowed a robust, efficient, and synchronized induction of gametocytogenesis by expression of GDV1 upon addition of Shield-1 and therefore a solid basis for phenotypic analysis. First, using the C-terminal GFP tag, we confirmed expression of all four *Pf*ApiATs in gametocytes. As expected, most *Pf*ApiATs remain PPM localized during gametocytogenesis, which was additionally confirmed by the colocalization with the episomally expressed PPM marker Lyn (39)-mCherry (38) (Fig. 4A to F). The exception was *Pf*ApiAT9, which showed only a faint background staining in all gametocyte stages in the 3D7-ApiAT9-GFP line (Fig. 4D). The observed prominent GFP signal at the food vacuole in gametocytes is most likely an unspecific hemozoin signal and not derived from GFP fusion proteins, as also observed in 3D7-iGP and 3D7 wild-type control parasites (Fig. S4C). Of note, *Pf*ApiAT2 was observed to be strongest expressed in early stage gametocytes and weaker in late stages (Fig. 5B and D; Fig. S4D), while *Pf*ApiAT4 showed strongest expression in late stage gametocytes (Fig. 5C and E). Next, we investigated the consequence of glmS-based conditional knockdown for *Pf*ApiAT2 and *Pf*ApiAT4 (Fig. 5A). Although 75 to 80% knockdown of *Pf*ApiAT2 or *Pf*ApiAT4 expression was achieved (Fig. 5B to E), no significant reduction in gametocytemia or aberrant gametocyte development could be detected (Fig. 5F). This was reinvestigated by targeted gene disruption. Likewise, deletion of these two genes as well as of *Pf*ApiAT8 and *Pf*ApiAT10 did not result in any measurable impairment of gametocyte development or morphology, indicating the dispensability of these individual *Pf*ApiATs for the sexual stage development of the parasite (Fig. 6 and Fig. S5).

10.1128/mSphere.00743-21.4 FIG S4 PCR analysis of 3D7-iGP-ApiAT-GFP cell lines and Giemsa smears from *Pf*ApiAT2 and *Pf*ApiAT4 conditional knockdown experiments. (A) PCR-based analysis of unmodified wild-type (WT) and transgenic knock-in (KI) cell lines (3D7-iGP-ApiAT2-GFP-glmS, 3D7-iGP-ApiAT4-GFP-glmS, 3D7-iGP-ApiAT8-GFP, and 3D7-iGP-ApiAT10-GFP). (B) Giemsa smears of stage I to V 3D7-iGP-ApiAT2-GFP-glmS and 3D7-iGP-ApiAT4-GFP-glmS gametocytes cultured either without (control) or with 2.5 mM glucosamine (GLCN). Bar, 5 μm. (C) Live-cell imaging of 3D7-iGP and 3D7 parasites. White arrowheads indicate remaining GDV1-GFP signal observed in close proximity to the nucleus indicated by the Hoechst signal. The stages are indicated. Nuclei were stained with Hoechst-33342. Bar, 2 μm. (D) Live-cell microscopy of 3D7-iGP-ApiAT2 stage IV and V gametocytes from Fig. 5B, with adjusted brightness. Bar, 2 μm. Download FIG S4, JPG file, 1 MB. Copyright © 2021 Wichers et al. 2021 Wichers et al. https://creativecommons.org/licenses/by/4.0/ This content is distributed under the terms of the Creative Commons Attribution 4.0 International license.

10.1128/mSphere.00743-21.5 FIG S5 PCR analysis of 3D7-iGP-ApiAT-TGD cell lines and Giemsa smears from 3D7-iGP-ApiAT8-TGD stage IV and V gametocytes showing normal morphology. (A) PCR-based analysis of unmodified wild-type (WT) and transgenic TGD cell lines (3D7-iGP-ApiAT2-TGD, 3D7-iGP-ApiAT4-TGD, 3D7-iGP-ApiAT8-TGD, and 3D7-iGP-ApiAT10-TGD). (B) Giemsa smears of 3D7-iGP-ApiAT8-TGD and 3D7-iGP stage IV and V gametocytes from three independent experiments. Bar, 5 μm. Download FIG S5, TIF file, 1.6 MB. Copyright © 2021 Wichers et al. 2021 Wichers et al. https://creativecommons.org/licenses/by/4.0/ This content is distributed under the terms of the Creative Commons Attribution 4.0 International license.

## DISCUSSION

We localized four putative amino acid transporters (*Pf*ApiAT2, *Pf*ApiAT4, *Pf*ApiAT8, and P*f*ApiAT10) of the ApiAT family to the PPM in asexual blood stage parasites and gametocytes. Due to the low expression of *Pf*ApiAT9-GFP—in agreement with the transcript levels of *apiat9* in these stages (37, 42, 43)—no conclusive localization could be delivered. The observed PPM localization of the investigated ApiATs is in concordance with the following published data. (i) The P. berghei ApiAT8 homologue was shown to be a general cationic amino acid transporter of the PPM (29, 30). (ii) *Pf*ApiAT8 has recently been localized to the PPM using an overexpression approach (31). (iii) Several T. gondii ApiATs (*Tg*ApiAT1, *Tg*ApiAT2, *Tg*ApiAT3-1, *Tg*ApiAT3-2, *Tg*ApiAT3-3, *Tg*ApiAT5-3, *Tg*ApiAT6-1, and *Tg*ApiAT6-3) have been located at the PPM as well (25, 29, 32, 33, 44).

Recent work using episomally overexpressed *Pf*ApiAT10-GFP implied an association of this transporter with the mitochondrial membrane (36) in P. falciparum Dd2 (*Pf*Dd2) parasites. This localization differs from the observed PPM localization of endogenously GFP-tagged *Pf*ApiAT10 in both 3D7 as well as 3D7-iGP parasites (Fig. 1B and 4C; see also Fig. S2A in the supplemental material) reported in this study. We confirmed the PPM localization of *Pf*ApiAT10 by its overexpression either as a GFP or mCherry fusion using two different promoters (*crt* [45] and *ama1* [46]). In line with that, the reported reduced sensitivity to the mitochondrial electron transport chain inhibitor atovaquone (47) upon knockout of *Pf*ApiAT10 (36) was not observed upon targeted gene disruption in our study (Fig. S2B). It is possible but appears unlikely that the reported mitochondrial association as well as reduced sensitivity to atovaquone upon overexpression is due to the different parasite strains (*Pf*Dd2 [48] versus 3D7 [this study]), given that *PfDd2_apiat10* (PFDd2_030017500) has only one silent mutation at position G1080A compared to *Pf3D7_apiat10* (49). Of note, *Pf*ApiAT10 is also not part of the recently published *Plasmodium* mitochondrial proteome (50).

During the intraerythrocytic development of the parasite, the amino acid requirements are largely covered by degradation of the globin polypeptide (14, 51), although—for instance—the import of isoleucine is crucial for the survival of the parasite, as P. falciparum lacks the canonical pathways for its biosynthesis (52) and adult human hemoglobin lacks this amino acid. Dedicated amino acid transporters could fill this gap. Therefore, we tested the impact of functional inactivation of individual *Pf*ApiATs on parasite growth in asexual and sexual blood stages of P. falciparum. We observed only a minor but significant reduction in parasite growth upon knockdown or gene disruption of *Pf*ApiAT2 and *Pf*ApiAT4 in asexual blood stages without compensatory upregulation of other *Pf*ApiATs on the transcriptional level, as indicated by qPCR analysis. The phenotypes are in agreement with a previously reported reduced parasite multiplication rate of 36% in *Pb*ApiAT4 knockout parasites (35). Moreover, our data are also in line with the finding that *Pb*ApiAT8 is not essential for asexual replication (29, 30, 35, 53). Additionally, functional inactivation of individual *Pf*ApiAT did not result in parasites more sensitive to reduced amino acid concentrations in the medium. The absence of profound growth effects might be explained by functional redundancy either within the ApiAT family, as recently observed for T. gondii (34), or by the presence of yet unassigned transporters capable of transporting essential amino acids such as isoleucine across the PPM. Like in T. gondii, overlapping substrate specificities and lower transport levels might be sufficient for parasite growth *in vitro* (34). Of note, in T. gondii, the arginine transporter *Tg*ApiAT1 has been shown to be differently regulated on the translational level in dependence of arginine mediated by an upstream open reading frame (uORF) present on the 5′ leader sequence of the transcript (26). A similar layer of regulation might also be present in *Pf*ApiATs. However, since *Toxoplasma* and *Plasmodium* share only one ApiAT, the likely most ancestral ApiAT2 (25), the regulatory elements as well as the general characteristics and substrates of the different *Pf*ApiATs remain largely unknown.

Interestingly, a knockout of the ApiAT8 homologue of P. berghei (PBANKA_0208300) resulted in strongly reduced number of mature gametocytes with an aberrant morphology of the remaining parasites (30) and strongly reduced exflagellation (35). In our study, functional inactivation of *Pf*ApiAT8 via targeted gene disruption had no impact on gametocyte development and morphology, which might reflect the pronounced differences in gametocyte development between the rodent-infecting P. berghei and the human-infecting P. falciparum parasites (54). For future work, it will be interesting to target multiple ApiATs by gene disruption in parallel to assess their putative synergy and to probe into *Pf*ApiAT function in other fast-replicating stages of P. falciparum such as liver stages, for which the essentiality of several metabolic processes has recently been shown and that primarily rely on the amino acid uptake from their host (55).

## MATERIALS AND METHODS

### P. falciparum culture.

Blood stages of P. falciparum 3D7 (56) were cultured in human red blood cells (O+ or B+). Cultures were maintained at 37°C in an atmosphere of 1% O_2_, 5% CO_2_, and 94% N_2_ using RPMI complete medium containing 0.5% Albumax according to standard protocols (57). In order to obtain highly synchronous parasite cultures, late schizonts were isolated by Percoll gradient (58) and cultured with fresh erythrocytes for 4 h. Afterwards sorbitol synchronization (59) was applied in order to remove remaining schizonts resulting in a highly synchronous ring stage parasite culture with a 4-h age window.

Induction of gametocytogenesis was done as previously described (41, 60). Briefly, GDV1-GFP-DD expression was achieved by the addition of 2 or 4 μM Shield-1 to the culture medium, and gametocyte cultures were treated with 50 mM *N-*acetyl-d-glucosamine (GlcNAc) for 5 days starting 72 h after Shield-1 addition to eliminate asexual parasites (61). Alternatively, asexual ring stage cultures with >10% parasitemia, cultured in the presence of choline, were synchronized with sorbitol (59) and washed twice in choline-free RPMI medium. Cells were kept in choline-free medium for the entirety of the assay. After one reinvasion cycle, cultures at trophozoite stage were treated with 50 mM GlcNAc (61) and kept on this for 5 days. Gametocytes were maintained in RPMI complete medium containing 0.25% Albumax and 0.25% sterile filtered human serum (Interstate Blood Bank, Inc., Memphis, TN, USA).

Growth assays in low-amino acid medium were performed using amino acid-restricted RPMI medium prepared as previously described (20). Briefly, complete medium was added in a 1/20 dilution to amino acid-free RPMI 1640 medium (catalog no. R9010-01; US Biological). This resulted in a 1:20 of the concentration of every amino acid compared to the standard complete RPMI-based medium.

### Cloning of plasmid constructs for parasite transfection.

For endogenous tagging using the SLI system (38) and glmS-based conditional knockdown (40), a 855-bp (*Pf*ApiAT4/MFR5/PF3D7_1129900) and 1,001-bp (*Pf*ApiAT2/MFR4/PF3D7_0914700) homology region was amplified using 3D7 guide DNA (gDNA) and cloned into pSLI-PIC1-GFP-glmS (62) using the NotI and MluI restriction sites.

For endogenous GFP tagging, a 886-bp (*Pf*ApiAT8/NPT1/PF3D7_0104800), 953-bp (*Pf*ApiAT9/MFR2/PF3D7_0104700), and 881-bp (*Pf*ApiAT10/MFR3/PF3D7_0312500) homology region was amplified using 3D7 gDNA and cloned into pSLI-GFP (38) using the NotI and MluI restriction sites.

For SLI-based targeted gene disruption (SLI-TGD), a 396-bp (*Pf*ApiAT4 and *Pf*ApiAT8), 402-bp (*Pf*ApiAT9), 393-bp (*Pf*ApiAT10), and 435-bp (*Pf*ApiAT2) homology region was amplified using 3D7 gDNA and cloned into the pSLI-TGD plasmid (38) using the NotI and MluI restriction sites.

For overexpression constructs, the full-length *Pf*ApiAT10 sequence was amplified from parasite gDNA and cloned into pARL-ama1 (46)-AIP-mCherry-yDHODH (62) using the XhoI and KpnI restriction sites or into the pARL-crt (45)-PF3D7_0324600-GFP-hDHFR (37) plasmid using the KpnI and AvrII restriction sites. Oligonucleotides used to generate the DNA fragments are summarized in Table S1 in the supplemental material.

10.1128/mSphere.00743-21.6 TABLE S1 Oligonucleotides used for cloning and quantitative real-time PCR (qPCR). Download Table S1, PDF file, 0.03 MB. Copyright © 2021 Wichers et al. 2021 Wichers et al. https://creativecommons.org/licenses/by/4.0/ This content is distributed under the terms of the Creative Commons Attribution 4.0 International license.

For colocalization experiments, the plasmid pLyn-FRB-mCherry (38) was used.

### Western blot analysis.

Immunoblots were performed using saponin-lysed, infected erythrocytes. Parasite proteins were separated on a 12% sodium dodecyl sulfate-polyacrylamide gel (SDS-PAG) as described previously (63, 64) and transferred to a nitrocellulose membrane (Amersham Protran; 0.45-μm-pore-size nitrocellulose membrane; GE Healthcare) using a Trans-Blot device (Bio-Rad) according to the manufacturer’s instructions. The membranes were blocked with 3% skim milk in Tris-buffered saline (TBS) for 30 min and then probed with mouse anti-GFP (clone 7.1 and 13.1; 1:1,000, Roche) or rabbit anti-aldolase (65) (1:2,000). The chemiluminescent signal of the horseradish peroxidase-coupled secondary antibodies (Dianova) was visualized using a Chemi Doc XRS imaging system (Bio-Rad) and processed with Image Lab 5.2 software (Bio-Rad).

To perform loading controls and ensure equal loading of parasite material, rabbit antialdolase (65) antibodies were used. The corresponding immunoblots were incubated twice in stripping buffer (0.2 M glycine, 50 mM dithiothreitol, 0.05% Tween 20) at 55°C for 1 h and washed three times with Tris-buffered saline for 10 min before reprobing.

### Transfection of P. falciparum.

For transfection, Percoll-purified (58) parasites at late schizont stage were transfected with 50 μg plasmid DNA using Amaxa Nucleofector 2b (Lonza, Switzerland) as previously described (66). Transfectants were selected using either 4 nM WR99210 (Jacobus Pharmaceuticals), 0.9 μM DSM1 (67) (BEI Resources), or 2 μg/ml blasticidin S (Life Technologies, USA). In order to select for parasites carrying the genomic modification via the SLI system (38), G418 (ThermoFisher, USA) at a final concentration of 400 μg/ml was added to a culture with about 5% parasitemia. The selection process and integration test were performed as previously described (38).

### Imaging.

All fluorescence images were captured using a Zeiss Axioskop 2plus microscope with a Hamamatsu digital camera (model C4742-95) or a Leica D6B fluorescence microscope equipped with a Leica DFC9000 GT camera and a Leica Plan Apochromat 100×/1.4 oil objective.

Microscopy of live-parasite-infected erythrocytes was performed as previously described (68). Briefly, parasites were incubated in standard culture medium with 1 μg/ml Hoechst-33342 (Invitrogen) for 15 min at 37°C prior to imaging. Infected erythrocytes (5.4 μl) were added on a glass slide and covered with a cover slip. Nuclei were stained with 1 μg/ml Hoechst-33342 (Invitrogen). Mitochondria were visualized by incubation of parasites with 20 nM MitoTracker Red 665 CMXRos (Invitrogen) for 15 min at 37°C prior to imaging. Contrast and intensities were linear adjusted for clarification and cropped images were assembled as panels using Fiji (69) and Adobe Photoshop CC 2021.

### Growth assay.

For growth assays of TGD cell lines, a flow cytometry assay, adapted from previously published assays (70, 71), was performed to measure proliferation over 5 days. For growth under low-amino acid conditions, TGD and wild-type cell lines were cultured in parallel in standard and amino acid-depleted medium for 5 days. Each day parasite cultures were resuspended, and 20-μl samples were transferred to an Eppendorf tube. Eighty microliters of RPMI medium containing Hoechst-33342 and dihydroethidium (DHE) was added to obtain final concentrations of 5 μg/ml and 4.5 μg/ml, respectively. Samples were incubated for 20 min (protected from UV light) at room temperature, and parasitemia was determined using an LSRII flow cytometer by counting 100,000 events using the FACSDiva software (BD Biosciences) or using an ACEA NovoCyte flow cytometer.

### Gametocyte quantification assay.

Giemsa-stained blood smears at day 10 postinduction of GDV1 expression were obtained, and at least 10 fields of view were recorded using a 63× objective per treatment and time point. Erythrocyte numbers were then determined using the automated Parasitemia software (http://www.gburri.org/parasitemia/), while the number of gametocytes was determined manually in >700 erythrocytes per sample.

### glmS-based knockdown.

The glmS-based knockdown assay was adapted from previously published assays (40, 72). To induce knockdown, highly synchronous early ring stage parasites were split in two dishes, 2.5 mM glucosamine was added to one of them, and parasite growth was measured by fluorescence-activated cell sorting (FACS) after two and four parasite replication cycles. Parasite cultures were inspected daily by Giemsa smears and, if necessary, diluted to avoid growth bias caused by high parasitemia. As an additional control, the same amount of glucosamine was also added to 3D7 wild-type parasites. For all analyses, medium was changed daily, and fresh glucosamine was added every day.

Knockdown was quantified by fluorescence live-cell microscopy using schizonts about 40 h after glucosamine treatment. Parasites of similar sizes were imaged, and fluorescence was captured with the same acquisition settings to obtain comparable measurements of the fluorescence intensity. Fluorescence intensity (integrated density) was measured with Fiji (69), and background was subtracted in each image. The data were visualized with GraphPad Prism version 8 (GraphPad Software, USA).

For knockdown experiments in gametocytes, synchronized ring stage cultures were induced by the addition of Shield-1 as described above. At day 3 postinduction, the culture was spilt into two dishes, and one dish was cultured in the presence of 2.5 mM glucosamine for the remaining 10 days. Knockdown was quantified by fluorescence live-cell microscopy at day 7 and 10 postinduction as described above.

### Drug assays.

Drug assays were adapted from previously described assays (73–75). Briefly, 3D7-iGP and 3D7-iGP-ApiAT10-TGD parasites were synchronized to a 4-h time window resulting in 0- to 4-h ring stage parasites. At 24 hpi, parasitemia was determined by flow cytometry, and the drug susceptibility assays were set up in black 96-well microtiter plates (Thermo Scientific) with 0.1% starting parasitemia and 2% hematocrit in a final volume of 200 μl. In each plate, infected erythrocytes in the absence of drugs treated with dimethyl sulfoxide (DMSO) only served as positive controls, while uninfected red blood cells (RBCs) served as negative controls (for background subtraction). Parasites were incubated with various concentrations of dihydroartemisinin (DHA) (catalog no. AG-CN2-0468; Adipogen, Switzerland) (0 to 50 nM) and atovaquone (catalog no. 23802; Cayman) (0 to 16 NM).

After 96 h of incubation, parasite growth was determined by measuring the fluorescence of SYBR Gold (Invitrogen). Therefore, 100 μl/well supernatant was discarded without disturbing the RBC layer and 100 μl of lysis buffer (20 mM Tris, 0.008% saponin, 0.08% Triton X-100, 1× SYBR Gold) was added to each well. Plates were incubated in the dark for 2 h at room temperature before measuring fluorescence using the EnVision Multimode plate reader (PerkinElmer) as described previously (75). In order to calculate 50% infective concentration (IC_50_) values, the measured values were normalized to the uninfected erythrocytes and plotted in GraphPad Prism version 8 (GraphPad Software, USA) as a percentage of the value for the DMSO control. Dose-response curves were generated using nonlinear regression (curve fit > dose-response inhibition > (log) inhibitor versus normalized response—variable slope).

### Quantitative real-time PCR.

Parasites at different time points (8, 16, 32, and 44 hpi) were harvested for 3D7-ApiAT2-TGD, 3D7-ApiAT4-TGD, 3D7-ApiAT8-TGD, 3D7-ApiAT10-TGD, and 3D7-WT to obtain RNA samples for quantitative real-time PCR (qPCR). Highly synchronous ring stage parasite cultures were grown for another 40 h, and TRIzol samples were harvested in the following cycle. Volumes of prewarmed TRIzol used for infected erythrocyte lysis and storage of RNA samples depended on the parasite stage: ring stages were lysed in 5× volumes, trophozoites in 10× volumes, and schizonts in 20× volumes of the settled cell pellet. RNA was purified and checked for the absence of genomic DNA. cDNA synthesis with random hexamers, and quantitative real-time PCR was performed exactly as previously described (76). Primers for each of the *apiat* genes, for genes to control for parasite stages (*sbp1* [77], *tom22*, and *ama1*) and for housekeeping genes (*arginyl-tRNA synthetase* [76] and *fructose-bisphosphate aldolase* [78] genes) are listed in Table S1. Amplification efficiencies of the primer pairs were determined over 6 log_10_ dilutions of gDNA (10 ng to 0.0001 ng) and were shown to have similar values between 1.915 and 2.001 (Table S1). Expression of *apiat* genes and controls were analyzed in relation to expression of the *arginyl-tRNA synthetase* gene (normalizer).

### Software.

Schematic protein representations were designed using IBS (79), predicted protein domains were obtained from plasmodDB (80) inferred from TMHMM (81). Parasite icons were generated using BioRender (biorender.com). Statistical analyses were performed with GraphPad Prism version 8 (GraphPad Software, USA).
