# Supplementary figures and images for: Characterization of Apicomplexan Amino Acid Transporters (ApiATs) in the Malaria Parasite Plasmodium falciparum
Source: mSphere. 2021 Nov 10;6(6):e00743-21. doi: 10.1128/mSphere.00743-21 (PMC8579892; doi:10.1128/mSphere.00743-21)

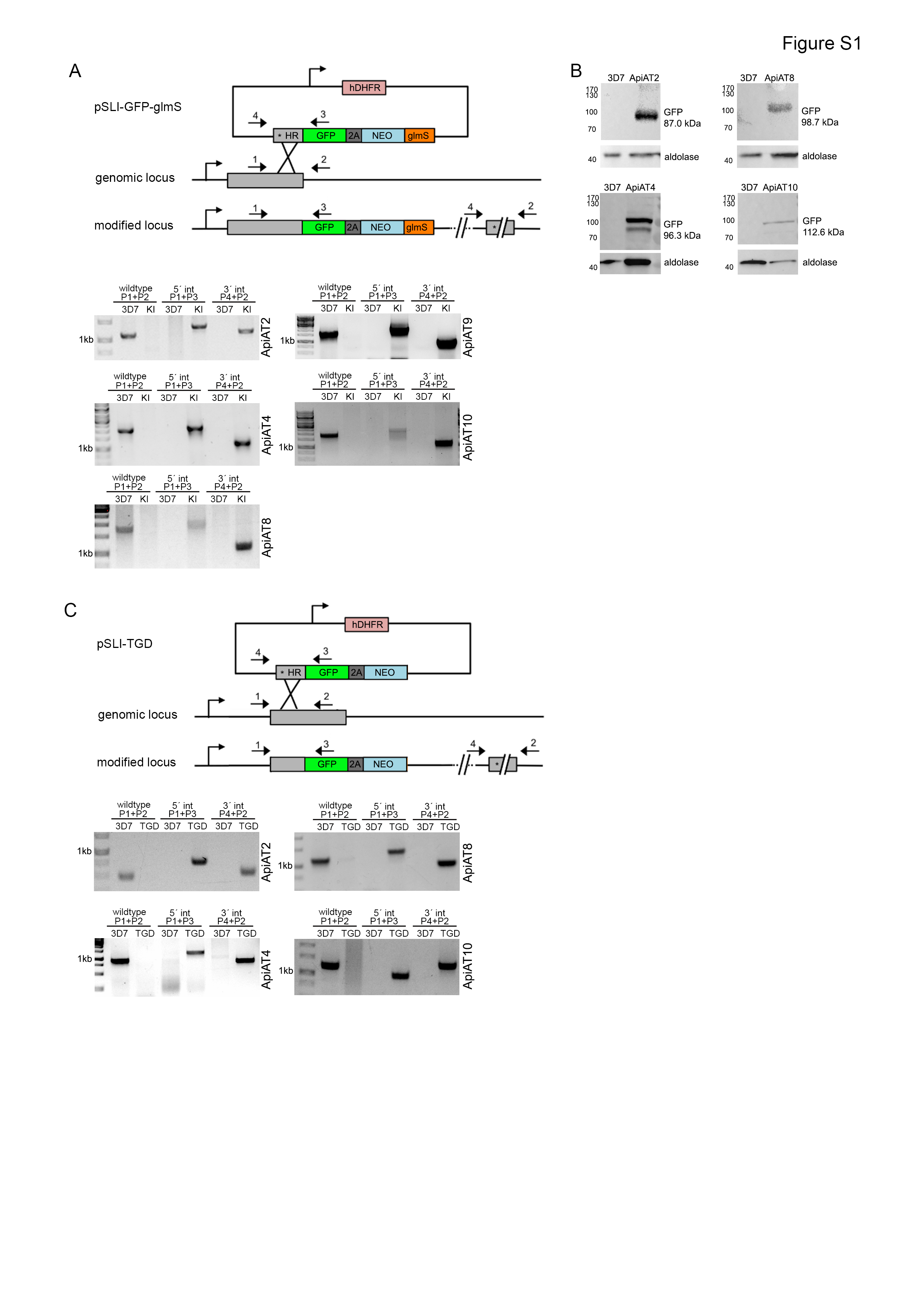

Supplement: FIG S1 [file msphere.00743-21-sf001.tif]

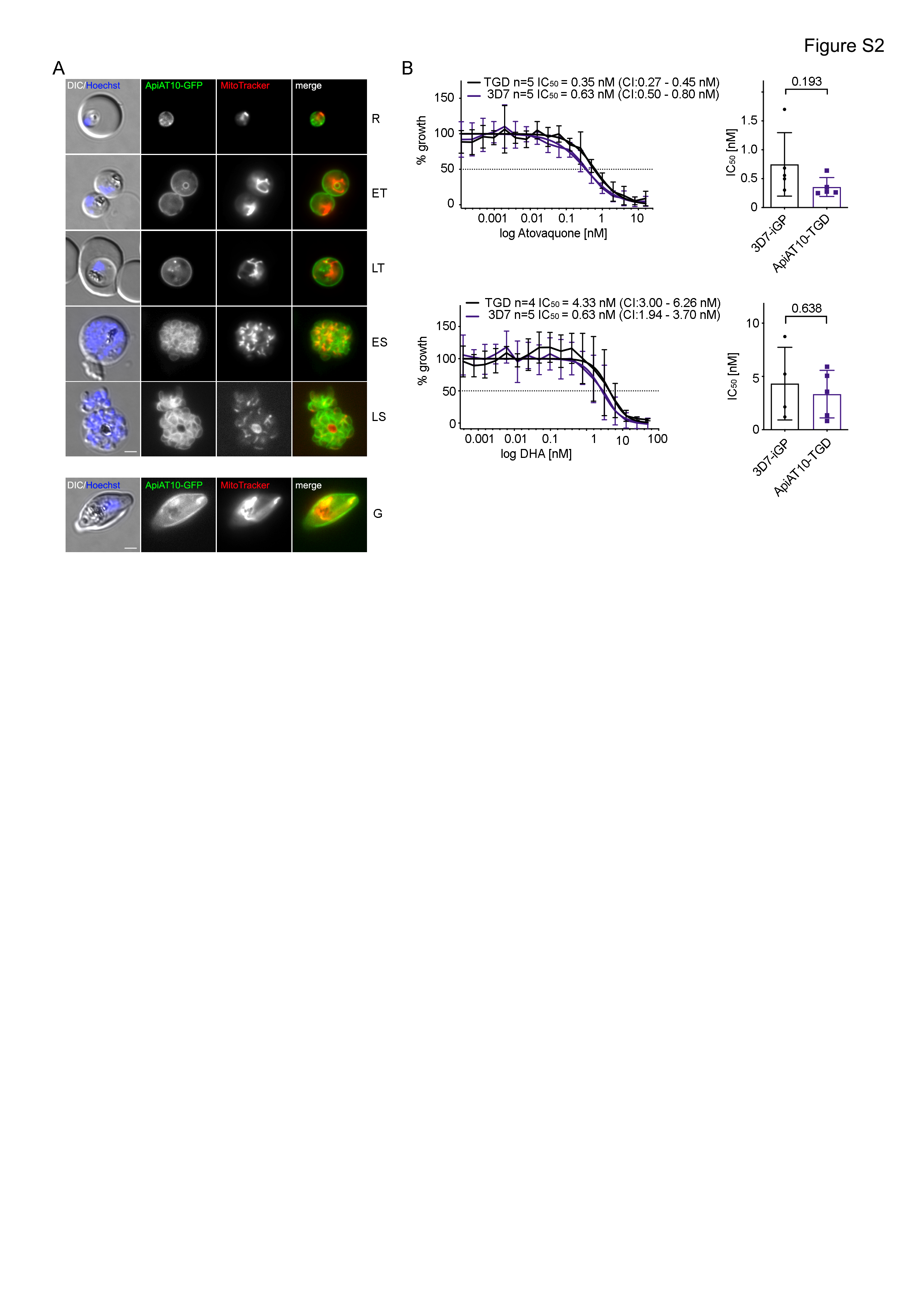

Supplement: FIG S2 [file msphere.00743-21-sf002.tif]

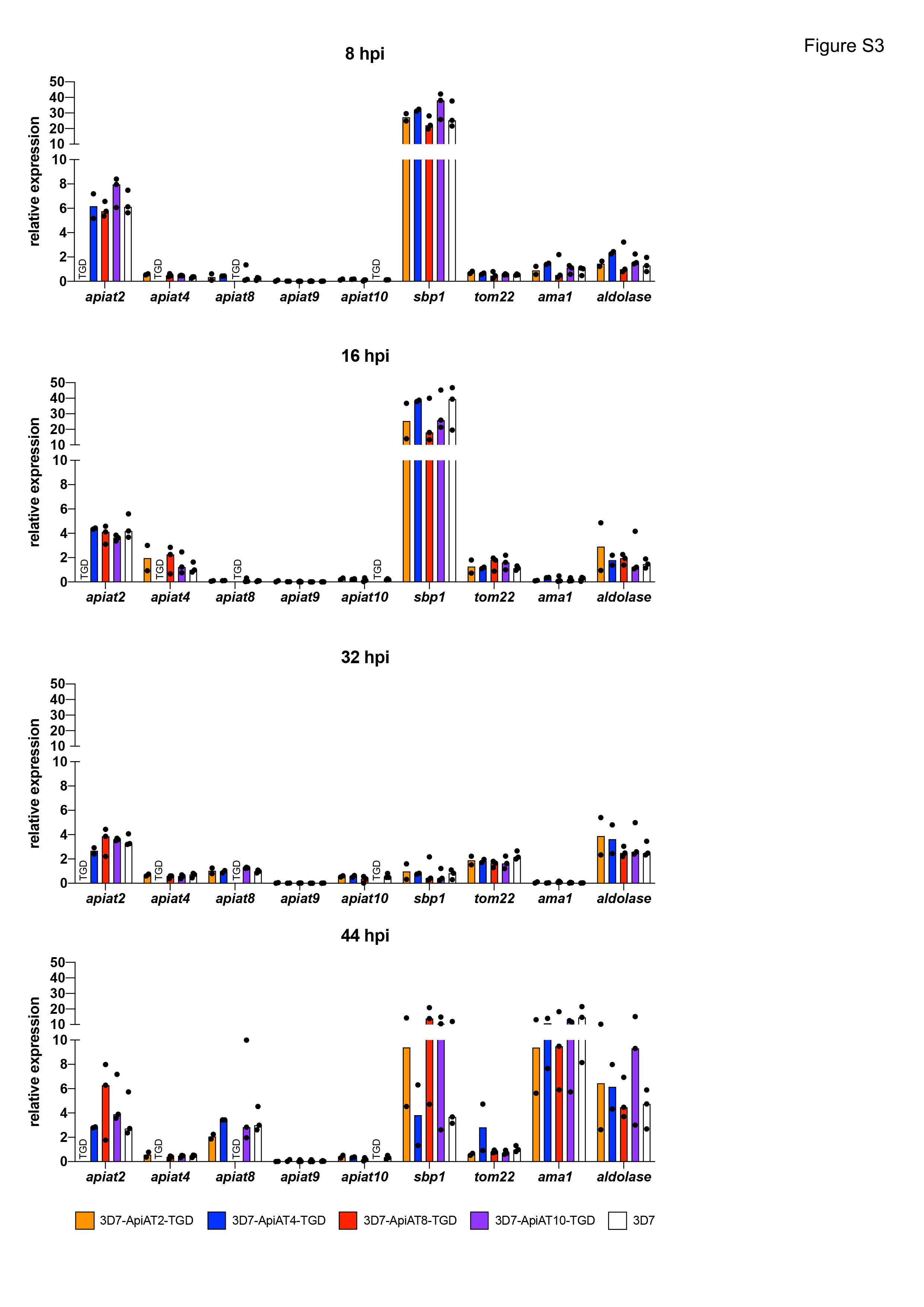

Supplement: FIG S3 [file msphere.00743-21-sf003.tif]

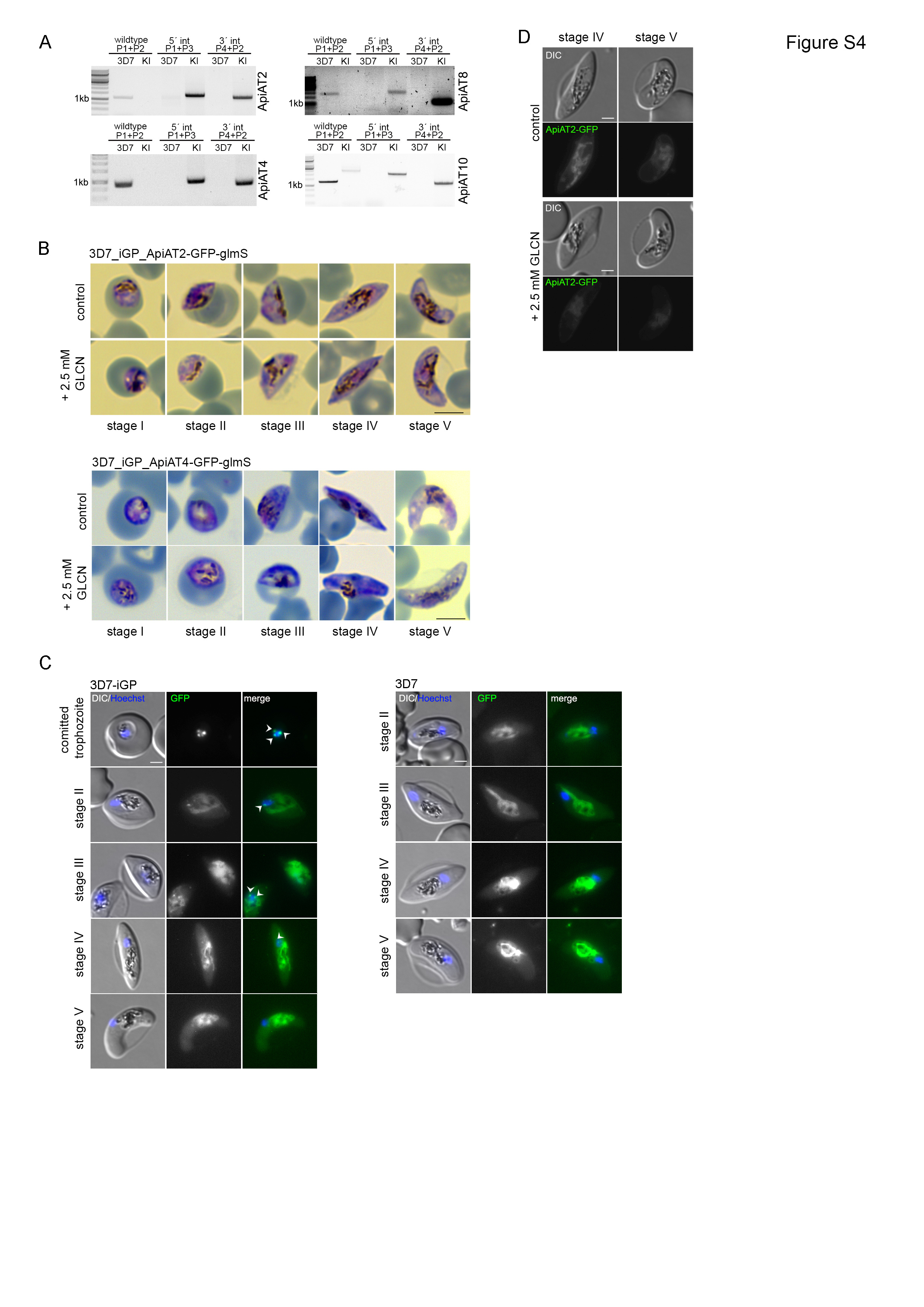

Supplement: FIG S4 [file msphere.00743-21-sf004.jpg]

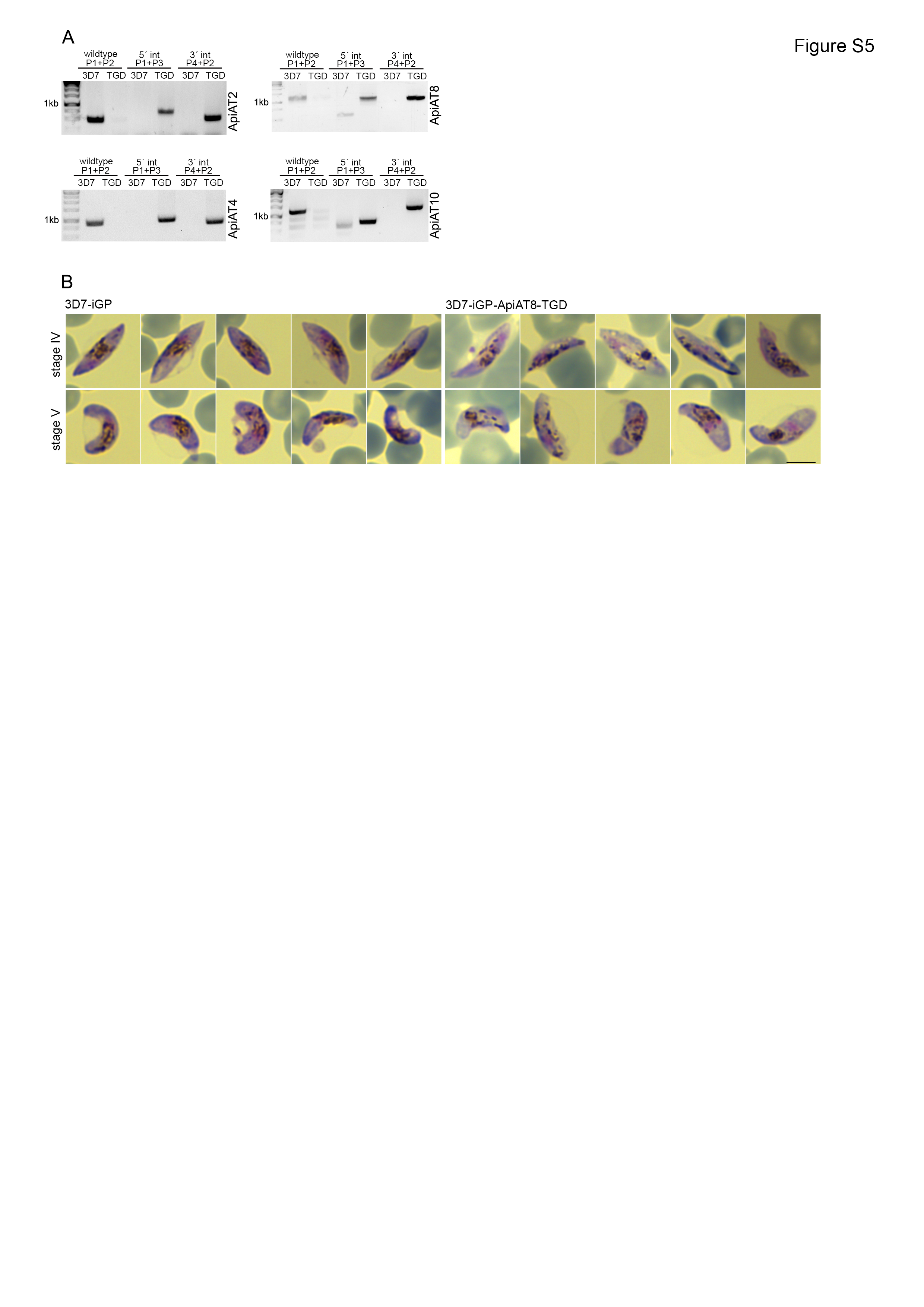

Supplement: FIG S5 [file msphere.00743-21-sf005.tif]
