# Supplementary material for: Characterization of Apicomplexan Amino Acid Transporters (ApiATs) in the Malaria Parasite Plasmodium falciparum
Source: mSphere. 2021 Nov 10;6(6):e00743-21. doi: 10.1128/mSphere.00743-21 (PMC8579892; doi:10.1128/mSphere.00743-21)
Supplement: TABLE S1 [file msphere.00743-21-st001.pdf]

Table S1: Oligonucleotides used for cloning and quantitative real-time PCR (qPCR).

ID	Target or Primer name	Sequence	Efficiency	Purpose
286	HR ApiAT4 fw	GGGacgcgtCTTTGATGATGCGTCGTCTCCATAG		cloning pSLI-GFP
287	HR ApiAT4 rv	GGGgcggccgctaaGACCATGATAAGAAAAAAGATGGAC		
329	HR ApiAT8 fw	GGGgcggccgctaaGGTAGATAAAGATAGTACAGA		
330	HR ApiAT8 rv	GGGacgcgtATTCATTGGGAGTTTATCCTTTTCTCC		
331	HR ApiAT9 fw	GGGgcggccgctaaGCCAAATTATATATACTAATAGCTCAATG		
332	HR ApiAT9 rv	GGGacgcgtCTTTTTGGTTTGACATCTTTTGTAT		
333	HR ApiAT10 fw	GGGgcggccgctaaGTGTGGATGATATAAACAATTCATC		
334	HR ApiAT10 rv	GGGacgcgtCTTTGAAGAAGGAAGGGAAATCATGTGAT		
454	HR ApiAT2 fw	GGGgcggccgctaaGTTCAGCGAGATCTACTGGATTTGC		
455	HR ApiAT2 rv	GGGacgcgtGTTTTTGTTTTTCTTCTTTTCTGTAAC		
321	HR ApiAT4-TGD fw	GCGGCCGCTAAAGTTCCAAAAAAAAGTCTACAATTATATTG		cloning pSLI-TGD
322	HR ApiAT4-TGD rv	acgcgtATCATATTCTCCATGCTCATTTTTTTGACA		
466	HR ApiAT2-TGD fw	GGGgcggccgctaaGCGTCAGATGTATCAAAGGAAAATAT		
467	HR ApiAT2-TGD rv	GGGacgcgtAAAGGCTAAAGCTAAAATAATTTGTC		
340	HR ApiAT8-TGD fw	gcggccgctaaAGTAACTCAATATTCCATAAG		
341	HR ApiAT8-TGD rv	acgcgtCCAACAAATAATATTAAATAACTGCCC		
344	HR ApiAT10-TGD fw	gcggccgctaaGTAAAGGACAAAACAATAATCTCCC		
345	HR ApiAT10-TGD rv	acgcgtGACTAATGATATTTTATTCGTT		
303	intcheck ApiAT4 fw	CGAAAAGGGAGATGAGTTCAAATGATAATG		integration check PCR
304	intcheck ApiAT4 rv	TATATATATATATAACCCTTTCGTTACTGG		
359	intcheck ApiAT9 fw	GGATACTTATAGGATTACAAAAGGG		
360	intcheck ApiAT9 rv	GACGTGTTCCCTTGAACTAATCCACG		
361	intcheck ApiAT8 fw	AGTAACTCAATATTCCATAAG		
362	intcheck ApiAT8 rv	GTATGTATGTATAATTGTTCGATATGG		
363	intcheck ApiAT10 fw	GTAAAGGACAAAACAATAATCTCCC		
364	intcheck ApiAT10 rv	CATATATAAAGAGGTACGTAAGAATAG		
483	intcheck ApiAT2 fw	CCAGGACAAGAATCCTTAATTTTTGG		
484	intcheck ApiAT2 rv	CCTGTTCATATGATCGTACACGTCAG		
373	intcheck ApiAT4-TGD fw	CTTCAGTTGTGTTTATTTTTGGTAT		
374	intcheck ApiAT4-TGD rv	CATTTCCTGGAAATAATCCTGATGC		
379	intcheck ApiAT8-TGD fw	GTTTACATTAAAAATTGTACTCTGTATG		
380	intcheck ApiAT8-TGD rv	CTATCTTTATCTACCATTCCGCTTGC		
383	intcheck ApiAT10-TGD fw	CCTTGTTAATAATAAACATGAAAAA		
384	intcheck ApiAT10-TGD rv	CCCCAAATACATTATTTTCATATATG		
485	intcheck ApiAT2-TGD fw	ACTTCATTTTATTCCTTTTTG		
486	intcheck ApiAT2-TGD rv	CCATACGTATGAGAATTTTAAAATGGC		
226	GFP rv	TTTTGTTGATAATGGTCTGC		
	GFP as 272	CCTTCGGGCATGGCACTC		
238	pARL sense 55	GGAATTGTGAGCGGATAACAATTTCACACAGG		
456	456_ApiAT2_for	GTCAAGCACCCAACGACCTA	1.949	qPCR
457	457_ApiAT2_rev	TGCCATCCACTAAATCCACCA		
458	458_ApiAT9_for	GGGATGAAAAATCTTTCCAATTCGT	1.961	
459	459_ApiAT9_rev	AGTTTATGTGCCTCCATGGTAA		
460	460_ApiAT4_for	TCAGCATGTGCAAATGGACAATTA	2.001	
461	461_ApiAT4_rev	CCACACCACACACTAGGTTCA		
462	462_ApiAT10_for	AGGCAATAGCTCAAGGGCTC	1.962	
463	463_ApiAT10-rev	TGAAGAAGGAAGGGAAATCATGTG		
464	464_ApiAT8_for	ACCAGGTGCCAAACAAAAGAC	1.982	
465	465_ApiAT8_rev	TATATGCACGCTGAGGTCGC		
	arginyl-tRNA synthetase_for (1)	TTCAAAACACGAAGTGGAACAAC	1.915	
	arginyl-tRNA synthetase_rev (1)	AATTCTCTGCAGCAAGTCGC		
	fructose-bisphosphate aldolase_for (2)	TGTACCACCAGCCTTACCAG	1.938	
	fructose-bisphosphate aldolase_rev (2)	TTCCTTGCCATGTGTTCAAT		
	sbp1_for (3)	TTAGCCGACGAACCAACACA	1.916	
	sbp1_rev (3)	TTCGGTTGTCTCTGGTACTGCA		
	tom22_for	GCCCATAAGGATGCCATTCG	1.961	
	tom22_rev	CACCTGCTATCCATACAACCCA		
	ama1_for	TGGGTAATCCATGGACGGAA	1.967	
	ama1_rev	TGAGTTCCAGCTACTTCAGCA		
JSW 75	ApiAT10 crt fw	GGGggtaccATGAAAAAAGTAAAGGACAAAAC		cloning pARL-*crt* ApiAt10-GFP
JSW 76	ApiAT10 crt rv	GGGcctaggCTTTGAAGAAGGAAGGGAAATCATG		
JSW 53	ApiAT10 ov fw	GGGctcgagATGAAAAAAGTAAAGGACAAAAC		cloning pARL-*ama1* ApiAt10-GFP
JSW 54	ApiAT10 ov rv	GGGggtaccCTTTGAAGAAGGAAGGGAAATCATG		

References

1. Bachmann, A. *et al.* Controlled human malaria infection with *Plasmodium falciparum* demonstrates impact of naturally acquired immunity on virulence gene expression. *PLoS Pathog.* **15**, e1007906 (2019).
2. Salanti, A. *et al.* Selective upregulation of a single distinctly structured *var* gene in chondroitin sulphate A-adhering *Plasmodium falciparum* involved in pregnancy-associated malaria. *Mol. Microbiol.* **49**, 179–191 (2003).
3. Petter, M. *et al.* Expression of *P. falciparum var* genes involves exchange of the histone variant H2A.Z at the promoter. *PLoS Pathog.* **7**, e1001292 (2011).
